# Healthcare Professionals’ Specialists’ Perception of Telemedicine in Romania—A Quantitative Study of Beliefs, Practices, and Expectations

**DOI:** 10.3390/healthcare11111552

**Published:** 2023-05-25

**Authors:** Octavian Andronic, George E. D. Petrescu, Andrada Raluca Artamonov, Alexandra Bolocan, Daniel Rădăvoi, Mihai Bran, Ana Maria Alexandra Stănescu, Viorel Jinga, Ștefan Busnatu

**Affiliations:** 1 Faculty of Medicine, Carol Davila University of Medicine and Pharmacy, 050474 Bucharest, Romania; octavian.andronic@umfcd.ro (O.A.); stefan.busnatu@umfcd.ro (Ș.B.); 2 University Emergency Hospital of Bucharest, Carol Davila University of Medicine and Pharmacy, 050098 Bucharest, Romania; 3 Bagdasar-Arseni Clinical Emergency Hospital, 041915 Bucharest, Romania; 4 Theodor Burghele Clinical Hospital, 061344 Bucharest, Romania; 5 Colțea Clinical Hospital, 030167 Bucharest, Romania

**Keywords:** telemedicine, telehealth, medical digitalization

## Abstract

Background: Telemedicine is the service of delivering medical care from a distance through the means of modern technology. It has many advantages, including improved access, decreased costs for both patients and clinics, more flexibility and availability, as well as more precise and individualized therapies. However, it is equally important to take into consideration all the challenges associated with this innovative way of providing care. This virtual technology has had an exponential growth, especially since the beginning of the COVID-19 pandemic, because it delivers great outcomes and suggests exciting future promises. Methods: The study involved the collection of responses from an online questionnaire comprising 26 questions that was distributed to healthcare professionals in Romania. Results: The questionnaire was completed by a number of 1017 healthcare professionals. We investigated and analyzed whether telehealth is seen as an important constituent of the healthcare system, if it is perceived as necessary, safe, well-managed by lawmakers, and easy to use, what advantages it has, what common practices specialists already employ and, additionally, the openness toward becoming more digitally educated for the purpose of streamlining the use of telemedicine. Conclusions: This paper reports on the perception of telemedicine among healthcare professionals in Romania, as constructive feedback represents an essential piece of the puzzle in assuring the smooth transition toward this facet of modern healthcare.

## 1. Introduction

Telemedicine represents the service of delivering medical care from a distance through the means of modern technology [1]. In a broader sense, telehealth comprises not only of the clinical aspect of healthcare but also of all the ways to efficiently communicate on matters of public health and medical education, as well as monitoring and supporting patients, within a digital climate [2].

Over time, it branched steadily into the majority of specialties, with a particular prominent role in radiology—a highly computerized field—but also in clinical fields such as family medicine, psychiatry and psychotherapy, internal medicine and its subspecialties [2,3]. This underlines its versatility, wide scope of practice and, ultimately, success, as it represents an increasingly growing area at the intersection of medicine, engineering, and computer science. Telehealth has had an exponential expansion, especially since the beginning of the COVID-19 pandemic [4,5], because it delivers good outcomes, similar to standard in-person interaction [2], and suggests exciting future promises, offering the possibility for isolated patients to receive medical care and improving the relationship between the patients and the healthcare professional [6]. Virtual medicine employs a wide variety of devices, ranging from the usual laptop or smartphone to a variety of cutting-edge wearables and sensors, which can assist the patient and prompt the physician toward an optimized treatment plan, in an extensive digital infrastructure, with the Internet as a pillar of strength [3,4,6,7].

There are multiple forms of telemedicine: (i) synchronous, bilateral, real-time interaction (most often through videoconference, but also available by telephone or written text); (ii) asynchronous specialist evaluation of specific medical information (such as radiologic interpretation or photographs of dermatologic lesions); (iii) monitoring and storage of health data—heart rate, blood pressure, blood sugar, oxygen saturation, ECG etc.—through various devices, for later assessment; (iv) assistance with low-acuity complaints and patient education on certain disorders and practices; (v) tracking conditions and behaviors; (vi) reminders for medication schedule, doses, future appointments; (vii) promoting good habits—diet, counting calories, exercise—and compliance; (viii) renewal of periodic prescriptions and even buying medication. Patients can employ a wide range of readily available, but sometimes costly technology, including computers, laptops, tablets, telephones, webcams, cameras, smartwatches and other wearables or sensors, smartphone applications and websites, frequently coupled with Internet connection. In addition, physicians need a medical platform with access to patient data and medical records, as well as digital documentation for each case [3,4,6,7]. Other important features of these computerized frameworks include the possibility of virtual pay for telemedicine bills as well as integrated feedback forms and transparency regarding the perceived quality of service [8].

Historically, the first step toward the goal of long-distance healthcare provision can be traced back six centuries in the past and is marked by the creation of the printing press, a pioneering device which allowed wider and faster dissemination of knowledge, including guidance on sanitation issues [9], which then led to a to a true information revolution in a wakening society, highlighting both the benefits of such a practice and the interest of the population. However, the first machine used with for actual medical reporting was the telegraph, which facilitated the care of wounded fighters in the American Civil War [10]. This kind of communication means paved the road for the use of telephone as an instrument for cooperation among physicians, patients and their families [9] and subsequently for radio assistance at sea and widespread television healthcare campaigns [10]. 

A pioneering example of modern telemedicine has been developed by NASA during the 1960s and targeted the health of astronauts by keeping specific parameters of their bodies and of the cabin environment under close observation, thus assuring the safety of space missions [11]. A decade later, ‘telemedicine’ was truly born in the collective mind by the publishing of a scientific paper about a successful medical collaboration between the Emergency Room of Massachusetts General Hospital and Logan International Airport Medical Station through television and telephone. The healthcare workforce comprised physicians and clinician nurses stationed at each site, respectively, and the patient population was represented by travelers and airport employees [12]. Later on, a similar initiative was aimed at rural communities with scarce healthcare resources and was also fruitful in preliminary results but, given the limited technologies available in that era, faced numerous issues in implementation and long-term sustainability [10], which have been the reasons why, over the following years, many other similar program were confronted with unfavorable reception and early cancellation [13]. 

Nevertheless, telehealth continued to evolve along with the continuous development of communication technology, both hardware- and software-wise, which culminated with the creation of the World Wide Web. Universal access, higher speed, decreasing costs—all played a fundamental role in a global, accelerating process of digitalization, with important reverberations in medicine, as well as in most fields of activity [9]. This process was increasingly visible over the last two decades, with the steady expansion of remote health services [2] backed by the ubiquity of computers and smartphones in the everyday life of general population [6]. Despite this, the true catalyzing force of an exponential growth in telemedicine has only emerged recently, over the COVID-19 pandemic, which was stimulated by a myriad of factors: social distancing measures and lockdowns, overburdened hospitals, infectious risk in clinics and even quarantined doctors [4,5]. This unprecedented modern crisis spiked the interest of patients, physicians and lawmakers in regard to remote healthcare, therefore promoting the quick progression of the digital and legal framework to support it [2]. With the tremendous excitement, favorable results and expected improvements, the use of telemedicine is estimated to only keep increasing [6].

Nowadays, this service has been adopted, to a certain degree, by most specialties, including surgical ones. However, over half of telehealth applications pertain to radiology [3], which, due to the intrinsic technologically-oriented nature of the field, has been the first to encourage and adopt the analog-to-digital transition [14,15]. Other well-suited areas include mental health, primary care, dermatology, internal medicine, cardiology, pulmonology, diabetes and nutrition, but it has also been well-developed in emergency settings, such as telestroke, teletrauma, teleburn and e-Intensive Care Unit [2,3]. Moreover, in emergency medicine, lower-cost solutions are being tested in order to decrease the burden of overcrowding the emergency departments [16]. In addition, telemedicine plays a key role in the direct collaboration among medical professionals, and it is most prolific in emergency medicine, radiology and pathology [1].

Telemedicine has many advantages, which include improved access to healthcare services, decreased costs for both patients and clinics, more flexibility and availability, as well as more precise and individualized therapies. It integrates all participants in healthcare and creates a wide network of coordinated medical services, facilitating collaboration and leading to increased satisfaction overall for both providers and recipients [1,4,6,8]. However, it is equally important to take into consideration all the challenges associated with this innovative way of providing medical care. Quality-wise, virtual consultations should be held up to the same standards as in-person. It has to be taken into account, however, that not all patients and not all disorders are suitable to the digital workflow, and further evidence is needed toward specific guidelines and protocols [1,6]. There are also legal concerns in regard to data security, privacy, and liability, which are still under careful scrutiny [17]. In addition, associated costs for the large-scale implementation and sustainability of these services might also be problematic and might lead to inequality and societal fragmentation [6,8].

This aim of this paper is to report on the perception of telemedicine among healthcare professionals (HCPs) in Romania, as constructive feedback represents an essential piece of the puzzle in assuring the smooth transition toward this facet of modern healthcare. Only being recently regulated by the Romanian government at the beginning of the pandemic [18], this technology is currently blooming and evolving in our country. Therefore, the beliefs and expectations of the experts who enact it are essential and play a key role in the development of virtual platforms, accessibility, patient education, future guidelines, and regulations. We investigated and analyzed whether telehealth is seen as an important constituent of the healthcare system, if it is perceived as necessary, safe, well-managed by lawmakers, easy to use and what advantages it has, what common practices specialists already employ and, additionally, the openness toward becoming more digitally educated for the purpose of streamlining the use of telemedicine. Therefore, the feedback presented in this paper would possibly contribute to further developing the field of telemedicine in Romania and countries with similar background and healthcare systems.

## 2. Materials and Methods

The study involved the collection of responses from a questionnaire that was distributed to HCPs (medical residents, specialty doctors, primary care physicians, nurses, pharmacists, and other allied healthcare providers) in Romania. The data were collected through an online survey (using a Google Form Questionnaire) between 1 March 2022 and 30 April 2022. The questionnaire was designed in order to obtain relevant information regarding the possible advantages and disadvantages of telemedicine. The dissemination of the questionnaire was carried out by electronic means, the target audience being HCPs. The questionnaire contained 26 questions, including 7 general questions (age, gender, city, medical degree, academic degree, type of engagement, specialty) and 19 specific Likert-scale questions (Table 1).

In order to be able to statistically evaluate the answers in relation to other monitored parameters (age, gender, job, position), we considered classifying the answers into two categories: agree (answers 4 or 5) and disagree (answers 1 or 2), considering that answer 3 is a neutral answer. One-way ANOVA was used to compare the means [19]. Statistical significance was considered at *p* value < 0.05.

## 3. Results

**Figure 1:**
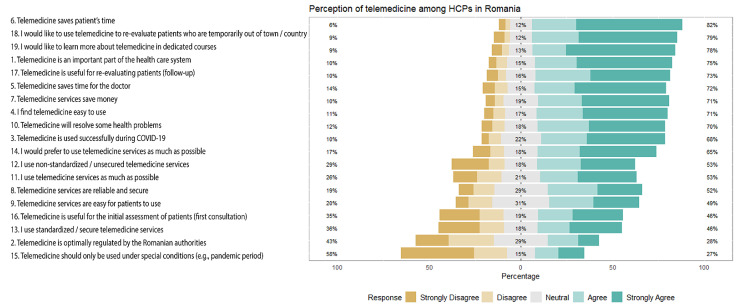
Questionnaire results. Percentages are summed up as follows: strongly disagree and disagree; neutral; agree and strongly agree. The questions are ordered based on the sum of “agree” and “strongly agree” respondents.

The questionnaire was completed by a number of 1017 respondents, with a national distribution, of which 78.4% were female and the mean (±SD) age being 44.9 years old (±12). Most of the respondents were doctors (85.5%), while 9.4% were nurses and 3.6% were pharmacists. All general data are available in Table 2. Results are summarized in Figure 1.

The majority of respondents (52.1%) strongly believe that telemedicine is an important part of the healthcare system. Regarding this statement, medical residents had statistically significant increased appreciation for the utility of telemedicine in the health care system, having a mean (±SD) of 4.39 (±0.83) when compared to senior or junior consultants who had a mean score of 4.06 (±1.2) (*p* = 0.003). Moreover, medical residents are the ones who overwhelmingly agree with this statement (87.8%). The breakdown by gender shows a higher percentage of men who disagree with this statement (16.8% vs. 8.0%) (*p* = 0.009).

There was a statistically significant difference regarding the successful implementation of telemedicine during the COVID-19 pandemic between the opinions of senior physicians, which had a mean (±SD) score of 4.05 (±1.15), and medical residents, where the mean (±SD) score was 3.82 (±1.12) (*p* = 0.036). 

Medical residents would be inclined to use more of the services offered by telemedicine for the follow-up of patients who temporarily live in another city, scoring a mean (±SD) of 4.45 ± 0.83 compared to more senior physicians 4.14 (±1.2) (*p* = 0.005). 

Another interesting finding was regarding the willingness to learn more about telemedicine through specialized courses, where we saw a statistically significant difference between the medical residents who scored a mean (±SD) of 4.55 (±0.85) when compared to senior and junior consultants, 4.15 (±1.2) (*p* < 0.0001).

Comparison between the perception of telemedicine between the senior physicians and medical residents is summed up in Table 3.

## 4. Discussion

Telemedicine is a branch of healthcare-related services concerned with providing medical assistance remotely by using modern technology [1]. In an expanded definition, telehealth refers not only to the purely clinical angle but also to pharmacy, nursing, and the broad communication of public health themes and medical education for both patients and healthcare workers, constant observation and support of the sick, all in the midst of extensive digitalization [2,3].

The advantages of telehealth are beyond dispute, as evidence shows that the outcomes are similar to traditional, in-person services (Table 4) [2]. One of the most important benefits of this technology is the possibility of reaching isolated areas, where medical facilities are scarce and non-specialized, so that people living there can enjoy the same quality and complexity of care without the additional travel expenses and wasted time. The decrease in costs is also seen on a systemic level: streamlined workflows, easier follow-up visits after discharging, better recovery management and optimized outcomes overall, culminating in a decreased risk of hospitalization, re-admission and mortality. This is coupled with increased performance in the Emergency Department, where triage through telemedicine helps alleviate the pressure off the healthcare workers in overwhelming situations (such as a pandemic or various natural disasters), thus leading to less burnout and more sustainability. There is also a more organized and transparent schedule in outpatient clinics, with a lower rate of missed appointments, less documentation and more convenience and flexibility for all parties involved, leading to doctors being able to actually see more patients within the same timeframe without employing or expanding office space [1,4,6,8]. Furthermore, telemedicine might be a solution for increasing the access to better medical care (i.e., better oncological care) for remote or disadvantaged populations [20].

In addition, epidemiological risks are non-existent, which has been a grave point of concern during the COVID-19 pandemic, especially in vulnerable populations, such as the elderly or the immunocompromised [8]. This has been doubled by a reduction in the use of personal protective equipment [4], which allowed a redistribution of resources toward other important areas. There are additional ways in which communities at risk benefit from telehealth, and they include increased access for people with disabilities, imprisoned, or culturally isolated [8].

Another plus is the encouragement of personalized healthy behaviors, on an individual level [6], which could increase compliance by directly involving the patient in his own care. This is extremely useful for chronic patients with complex medication schemes. Moreover, patients can pick the provider best suited to their needs and have a customized experience, with a more direct line of communication with the physician, which increases satisfaction levels. They can be tracked in real time and have treatment plans changed accordingly, in a precise, tailored manner [1,6,8]. 

On the one hand, is it obvious that routine visits and follow-ups can be conducted remotely [1]. On the other hand, telehealth is a useful tool in hyper-specialized care, with an often very limited number of professionals throughout the country, as well as in expanding access to various clinical trials [2,8].

In addition, this technology stimulates team-based work, as medical records can be easily shared among the healthcare workers involved in the care of each patient. These coordinated efforts help primary care doctors receive expert information when needed and enable a comprehensive yet very complex perspective over all medical issues, leading to better care [8]. This inter-connected network can further include carers and guardians as well as legislators and stakeholders [1].

In the future, telemedicine is said to become integrated in the longitudinal management of patients, further expanding access to medical care while simultaneously decreasing the total costs of the system [6]. Another key feature to be implemented is Artificial Intelligence, which could handle some of the basic tasks of the physicians, decreasing menial and routine responsibilities and allowing them to focus on medical issues, potentially improving job satisfaction levels [8].

Despite the clear advantages, it is paramount to take note of all the challenges that this technology brings, as they are equally important to a well-functioning system. Concerns and skepticism span across multiple areas.

From a medical point of view, telehealth lacks cues from physical examination as well as the immediate availability of specific laboratory and imaging information. This raises questions about diagnosis accuracy, which, in turn, has a fundamental impact on therapy plans and future outcomes. It is important to realize that telemedicine falls under the same regulations and standards as regular practice and should be judged accordingly. In addition, telemedicine might not be suited for complex medical issues pertaining to multiple specialties, which should but cannot be taken into account simultaneously. It is also not suitable for certain conditions, which cannot be diagnosed by history and a limited clinical exam alone [1,6,21]. Another point of concern is that websites and applications offering medical solutions to minor problems may disrupt professional treatment coordination in the long term. As a consequence, it has been even been proposed that telemedicine appointments should only be episodic, under special circumstances, so as to not interrupt the continuity of primary care [6]. 

From a legal perspective, telemedicine has only been recently regulated in Romania, at the beginning of the COVID-19 pandemic [18], and consensus within the European Union has not yet been reached [17]. The privacy and security of medical data represent an absolute priority, as does informed consent. Encryption plays a fundamental role, and access to videoconference session, images and other information has to be thoroughly controlled. It is essential to create laws which protect both the sick and the medical professionals, and physicians should be conscientious about liability issues and malpractice insurance. Another point of discussion is the equitable reimbursement of these services [1,6,8].

From an ethical angle, it is extremely important that telemedicine maintains the same values as the in-person interaction, and that the doctor–patient connection is established with the same care and respect. Some experts argue that telemedicine is most efficient when the physician and the patient have met before and already share a mutually trusting relationship [6]. Apart from the screen barrier, the patient has to still be seen as human, and medicine should remain patient-centered in all its forms, which might be difficult in the absence of in-person interactions, where some non-verbal signals are inevitably lost [1,7]. Another issue is the assessment of an appropriate-enough cognitive function to allow participation in an online consultation, which could be the case in mental health patients, the elderly, and others [2]. In addition, physicians should ponder whether the quality of care is appropriate to each case and not fall under the trap of novelty and innovation for the sake of it. As we are still in a transition period, more evidence is definitely needed in order to establish guidelines and standards applicable to telehealth services [6]. A different challenge refers to the risk of telehealth to actually worsen societal inequality, instead of alleviating it, as some demographic factors have been linked to a higher level of reluctance toward this technology, such as being male, older, non-white, or from a low socio-economic background. In addition, in such a globalized society, language and culture should also be taken into account [2,8].

From a logistic point of view, both healthcare providers and patients need to have access to modern, up-to-date hardware and software, which brings an increased initial cost. A reliable infrastructure must be set in place, possibly still being a problem in rural communities—who, incidentally, could benefit most from telemedicine [8]. Moreover, all parties have to possess enough knowledge in order to be able to use this technology independently. There are concerns that disadvantaged populations, especially financially or educationally, might not be able to adapt to this new system [6]. A widespread screening of these factors within the general population is desirable if the large adoption of telehealth should be expected in the future [2]. Other episodic, but potentially significant problems include device disconnection, malfunction or failure [8].

Therefore, telemedicine should be considered an asset and a facet of modern medicine rather than a replacement for traditional care [1,6,8]. It is important to take note of both its advantages and disadvantages to continuously improve by following both evidence and constructive criticism.

As far as feedback from health professionals is concerned, the responses are overwhelmingly positive, both in the literature and in our study. We found that three-quarters (74.8%) of the participants see telemedicine as an important or very important part of our healthcare system. Moreover, 69.5% think that telemedicine will actually solve some problems within out healthcare system, which is an impressive percent, given the fact that it is only related to expectations. In turn, this should encourage stakeholders, legislators, researchers, and companies to further advance the technology toward new directions.

Seventy-one percent of the responders think that telemedicine has been successfully implemented during the COVID-19 pandemic, but only 14.3% believe that it should be limited to a healthcare crisis. Instead, a majority of 57.7% disagree with this statement, and it can be inferred that this majority intends to keep using e-health beyond this particular epidemiological context. This is further reinforced by the 64.7% who would prefer to use telemedicine services as much as possible and the 77.8% who are open to learning more about this innovative technology. However, 9.4% of responders cited a rather low desire to participate in courses or training sessions about telemedicine. This can be explained partially by our findings that there is a statistically significant difference between the willingness of senior physicians and medical residents to participate in such courses, senior physicians being more reluctant. It is important to further investigate whether this is a consequence of disinterest, previous negative experiences or an already high proficiency in digital matters.

The easiness of use has been positively appreciated by the majority (67.7%), with only 3.9% rating the lowest score. On the other hand, only 49.2% of healthcare providers consider that this technology is easy or very easy to use for the patients, with a bigger percent (30.9%) of neutral responses than is the case of professional use. Additionally, 6.9% think that telemedicine is very hard to use for patients. Even though this number seems low, efforts should be concentrated toward increasing digital awareness and literacy among general population in order to better reach the disadvantaged.

As far as time management is concerned, 71.7% of responders are of the opinion that telemedicine brings a certain level of convenience to the service provider, and 84.1% believe it brings a certain level of convenience to the patients. The opposite side keeps the same trend, with 6.8% of medical professional believing that telemedicine does not improve their time management but only 3.6% thinking that it is not beneficial to the patients’ time. 

In regard to legislation, consensus has not been reached: most responders remain rather moderate and neutral about it but with a tendency toward dissatisfaction. A little over half of the responders (51.7%) believe that telemedicine is rather trustworthy and safe with a significant neutral response rate (29%). It is essential to have digital security measures in place and to ensure awareness campaign and education in this regard not only for the general population but for medical practitioners as well. However, in relation to the actual software used, responses are worrying. Only 45.9% of survey participants use standardized and secure programs, while 35.9% do not, at least to a certain level. In turn, 53.4% employ non-standardized, possibly non-encrypted services, while only 20.1% firmly deny doing so. This is an alarming signal that hospitals and clinics have to re-enforce data protection infrastructure, to implement safe and accessible software for providers and patients, and to advance educational campaigns among healthcare workers in order for them not to engage in unsafe patient communication anymore. 

Of the responders, 71.5% consider that telemedicine saves money to a certain degree.

A majority of 79.4% of responders are willing to use telemedicine for patients temporarily located elsewhere in the country or in the world. Furthermore, it would be interesting to explore different roles for this technology, as seen from the eyes of medical providers. This could identify certain gaps and problems on a systemic level.

On the issue of using telemedicine for the initial consult, consensus has not been reached, and the medical community is divided on whether this is a good approach or not, but the trend points toward a perceived benefit. However, 73.5% agree that virtual consults are suitable for follow-ups.

As limitations of our study, we have to underline the fact that this is an online survey through which the identity of the responders has not been verified. In addition, 78.4% were female, and 73.1% were doctors, which are two demographic variables that might not paint an accurate description of the medical workforce as a whole.

### Related Work

Other authors also evaluated the utility of telemedicine by addressing surveys to HCPs. For example, Wali et al. conducted a recent study that included 53 healthcare professionals that responded to a survey which evaluated the usage of virtual clinics in Saudi Arabia [22]. Based on their findings, the authors conclude that the majority of the physicians consider that reduced access to technology and lack of technical knowledge are two factors that influence the usability of virtual clinical platforms. In addition, a large study assessing over 1000 respondents from the field of oncology showed that 93% of the participants think that adverse outcomes are rarely or almost never associated with telemedicine [23]. Moreover, most of the respondents also consider that for benign cases, the experience offered by telemedicine is similar to that offered by standard visits.

Future inquiries should focus on the specific ways in which telemedicine is integrated within practice and especially what barriers and limitations have been encountered, as well as potential solutions. In addition, it would be good to identify specific areas to focus on during training sessions and courses on telemedicine, such as security, legislation, insurance policies and even the psychology behind virtual consults for improved experiences in the future. There should be extensive feedback from non-physician medical personnel, such as nurses, pharmacists, psychotherapists, social workers and other kinds of therapists. Moreover, feedback from healthcare students is another point of interest in order to find methods to better prepare them for an increasingly digitalized future during the span on their careers.

## 5. Conclusions

Our paper presents the feedback from a large number of HCPs from Romania regarding their perception of telemedicine. Overall, more than half of the respondents believed that telemedicine is an important part of the healthcare system (and almost 90% of the medical residents), and the majority of the respondents would like to use telemedicine to re-evaluate patients that are temporarily out of the town. Medical residents showed an increased interest in participating in courses in order to learn more about telemedicine. Telemedicine is a versatile tool that can facilitate the interaction between HCPs and patients, as well as among HCPs, with the goal of improving patient care and reducing the increasing burden on the medical systems. While there are certain limitations (lack of human interaction, technology-dependent, legislative issues), the general perception of the HCPs is that telemedicine plays an important part in the medical system, being time effective and easy to use, and having the potential of solving some of the issues of the medical systems.

## Figures and Tables

**Table 1:** Specific Likert-scale questions that were addressed to HCPs (1—disagree; 5—agree).

1. Telemedicine is an important part of the healthcare system
2. Telemedicine is optimally regulated by the Romanian authorities
3. Telemedicine is used successfully during COVID-19
4. I find telemedicine easy to use
5. Telemedicine saves time for the doctor
6. Telemedicine saves the patient’s time
7. Telemedicine services save money
8. Telemedicine services are reliable and secure
9. Telemedicine services are easy for patients to use
10. Telemedicine will resolve some health problems
11. I use telemedicine services as much as possible
12. I use non-standardized/unsecured telemedicine services
13. I use standardized/secure telemedicine services
14. I would prefer to use telemedicine services as much as possible
15. Telemedicine should only be used under special conditions (e.g., pandemic period)
16. Telemedicine is useful for the initial assessment of patients (first consultation)
17. Telemedicine is useful for re-evaluating patients (follow-up)
18. I would like to use telemedicine to re-evaluate patients who are temporarily out of town/country
19. I would like to learn more about telemedicine in dedicated courses

**Table 2:** General description of the study group.

Age	
Mean	44.9
Median	45
SD	12
Min	22
Max	87
**Sex**	
Male	220 (21.6%)
Female	797 (78.4%)
**Medical degree**	
Nurse	96 (9.4%)
Resident doctor	120 (11.8%)
Junior consultant	294 (28.9%)
Senior consultant	456 (44.8%)
Pharmacist	37 (3.6%)
Other	14 (1.4%)
**Academic degree**	
None	846 (83.2%)
Assistant Professor	79 (7.8%)
Lecturer	41 (4%)
Associate Professor	30 (2.9%)
Professor	21 (2.1%)
**Job sector**	
Public	468 (46%)
Private	329 (32.4%)
Both	220 (21.6%)

**Table 3:** Comparison between the perception of telemedicine between the senior physicians and medical residents.

Question	Senior and Junior Consultants Mean ± SD	Medical Resident Mean ± SD	*p* Value *
1. Telemedicine is an important part of the healthcare system	4.06 ± 1.12	4.39 ± 0.83	0.003
2. Telemedicine is optimally regulated by the Romanian authorities	2.74 ± 1.22	2.48 ± 1.13	0.025
3. Telemedicine is used successfully during COVID-19	4.05 ± 1.15	3.82 ± 1.12	0.036
4. I find telemedicine easy to use	3.95 ± 1.12	3.90 ± 1.11	0.65
5. Telemedicine saves time for the doctor	3.95 ± 1.25	4.15 ± 1.13	0.10
6. Telemedicine saves the patient’s time	4.29 ± 1.02	4.38 ± 0.90	0.32
7. Telemedicine services save money	4.04 ± 1.12	4.01 ± 1.09	0.75
8. Telemedicine services are reliable and secure	3.50 ± 1.21	3.23 ± 1.08	0.021
9. Telemedicine services are easy for patients to use	3.47 ± 1.20	3.28 ± 1.08	0.09
10. Telemedicine will resolve some health problems	3.90 ± 1.18	4.05 ± 1.05	0.19
11. I use telemedicine services as much as possible	3.46 ± 1.37	3.20 ± 1.43	0.05
12. I use non-standardized/unsecured telemedicine services	3.37 ± 1.46	3.28 ± 1.51	0.49
13. I use standardized/secure telemedicine services	3.06 ± 1.52	3.03 ± 1.48	0.79
14. I would prefer to use telemedicine services as much as possible	3.73 ± 1.33	3.97 ± 1.13	0.07
15. Telemedicine should only be used under special conditions (e.g., pandemic period)	2.38 ± 1.44	2.41 ± 1.41	0.85
16. Telemedicine is useful for the initial assessment of patients (first consultation)	3.05 ± 1.48	3.15 ± 1.57	0.51
17. Telemedicine is useful for re-evaluating patients (follow-up)	4.01 ± 1.11	4.15 ± 1.05	0.20
18. I would like to use telemedicine to re-evaluate patients who are temporarily out of town/country	4.14 ± 1.16	4.45 ± 0.83	0.005
19. I would like to learn more about telemedicine in dedicated courses	4.15 ± 1.18	4.55 ± 0.85	<0.0001

* one-way ANOVA.

**Table 4:** Advantages and disadvantages of telemedicine.

Advantages	Disadvantages
Outcomes similar to traditional, in-person services	Lack of physical examination
Reaching isolated areas	Might not be suitable for complex medical issues
Decrease in healthcare services costs	May disrupt professional treatment coordination in the long term
Decreased risk of hospitalization, re-admission and mortality	Concerns regarding privacy and security of medical data
Can be a triage solution, reducing the overwhelmed Emergency Departments	Appropriate assessment of cognitive function is particularly challenging in patients with mental health issues or elderly
Reduced epidemiological risks	Might worsen the societal inequality
Increases compliance for chronic patients	Need for modern up-to-date hardware and software which brings an increased initial cost
Useful tool for highly specialized care with a limited number of experts	Potential device disconnection, malfunction or failure
Promotes teamwork and can involve carers, guardians, legislators and stakeholders	
Can be augmented with Articifial Intelligence	

## Data Availability

Data is available upon request.
